# Geraniol Pharmacokinetics, Bioavailability and Its Multiple Effects on the Liver Antioxidant and Xenobiotic-Metabolizing Enzymes

**DOI:** 10.3389/fphar.2018.00018

**Published:** 2018-01-25

**Authors:** Barbara Pavan, Alessandro Dalpiaz, Luca Marani, Sarah Beggiato, Luca Ferraro, Donatella Canistro, Moreno Paolini, Fabio Vivarelli, Maria C. Valerii, Antonietta Comparone, Luigia De Fazio, Enzo Spisni

**Affiliations:** ^1^Department of Life Sciences and Biotechnology, University of Ferrara, Ferrara, Italy; ^2^Department of Chemical and Pharmaceutical Sciences, University of Ferrara, Ferrara, Italy; ^3^Department of Pharmacy and Biotechnology, Alma Mater Studiorum, University of Bologna, Bologna, Italy; ^4^Department of Biological, Geological and Environmental Sciences, Alma Mater Studiorum, University of Bologna, Bologna, Italy

**Keywords:** geraniol, gut, bioavailability, pharmacokinetics, xenobiotic-metabolizing enzymes

## Abstract

Geraniol is a natural monoterpene showing anti-inflammatory, antioxidant, neuroprotective and anticancer effects. No pharmacokinetic and bioavailability data on geraniol are currently available. We therefore performed a systematic study to identify the permeation properties of geraniol across intestinal cells, and its pharmacokinetics and bioavailability after intravenous and oral administration to rats. In addition, we systematically investigated the potential hepatotoxic effects of high doses of geraniol on hepatic phase I, phase II and antioxidant enzymatic activities and undertook a hematochemical analysis on mice. Permeation studies performed via HPLC evidenced geraniol permeability coefficients across an *in vitro* model of the human intestinal wall for apical to basolateral and basolateral to apical transport of 13.10 ± 2.3 × 10^-3^ and 2.1 ± 0.1⋅× 10^-3^ cm/min, respectively. After intravenous administration of geraniol to rats (50 mg/kg), its concentration in whole blood (detected via HPLC) decreased following an apparent pseudo-first order kinetics with a half-life of 12.5 ± 1.5 min. The absolute bioavailability values of oral formulations (50 mg/kg) of emulsified geraniol or fiber-adsorbed geraniol were 92 and 16%, respectively. Following emulsified oral administration, geraniol amounts in the cerebrospinal fluid of rats ranged between 0.72 ± 0.08 μg/mL and 2.6 ± 0.2 μg/mL within 60 min. Mice treated with 120 mg/kg of geraniol for 4 weeks showed increased anti-oxidative defenses with no signs of liver toxicity. CYP450 enzyme activities appeared only slightly affected by the high dosage of geraniol.

## Introduction

**FIGURE 1:**
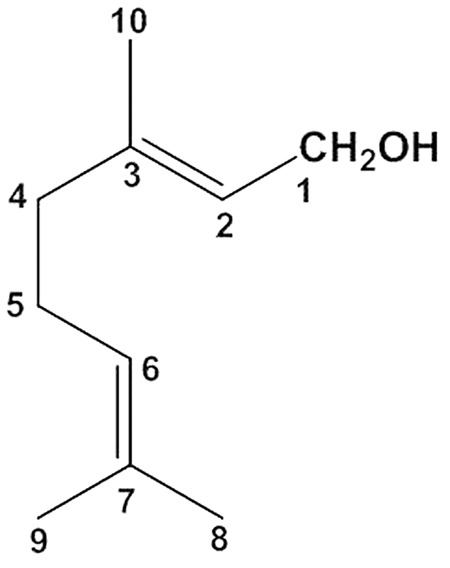
Chemical structure of geraniol.

Geraniol (3,7-dimethylocta-trans-2,6-dien-1-ol) is an acyclic monoterpene (**Figure 1**) with a water solubility of 100 mg/L at 25°C and an *n*-octanol/water partition coefficient of 2.65 (Turina et al., 2006). It is abundant in essential oils extracted from lemongrass, rose, lavender and other aromatic plants (Lapczynski et al., 2008; Chen and Viljoen, 2010). It is known to exert a wide spectrum of pharmacological activities, namely antimicrobial (Thapa et al., 2012), anti-inflammatory (Chen and Viljoen, 2010), antioxidant (Khan et al., 2013) and neuroprotective (Rekha et al., 2013) effects. For example, oral administration of geraniol as an antimicrobial agent effectively prevented colitis-associated dysbiosis and decreased the systemic inflammatory profile of colitic mice (De Fazio et al., 2016). Moreover, oral administration of geraniol supported the ability of dopaminergic neurons to survive free radical injury by inducing the production of antioxidant enzymes and reducing the expression of apoptotic markers (Rekha et al., 2014). These properties indicate that geraniol is a natural compound potentially able to prevent the progression of Parkinson’s disease and other neurodegenerative disorders (Rekha et al., 2014) and to serve as a therapeutic agent in the treatment of a wide range of cancers, including lung, pancreatic, hepatic and kidney tumors (Cho et al., 2016). The potential therapeutic effects of geraniol have major clinical implications as this essential oil component is classified in the generally-recognized-as-safe (GRAS) category (Lapczynski et al., 2008).

Relatively few data are available on geraniol hepatic metabolism and renal excretion. The initial step in the metabolism of geraniol seems to be its allylic hydroxylation at C8 yielding the alcohol intermediates that occur in rat liver and lung microsomes (Scheline, 1991). Conjugated or free geraniol metabolites were identified in the urine of rats after oral administration of the monoterpene (800 mg/Kg for 20 days). The most prominent acidic compounds were geranic acid, 8-carboxy geraniol, 3-hydroxycitronellic acid and Hildebrandt acid, while the neutral metabolites identified were geraniol itself and 8-hydroxygeraniol. These compounds are obtained by the oxidation of C1, C3, C7, and C8 geraniol groups (Chadha and Madyastha, 1984; Scheline, 1991). In liver, phase I (oxidation, reduction and hydrolysis) and phase II (conjugation) reactions assume the role of the enzymatic pathways mainly involved in xenobiotics metabolism (Raunio et al., 2015). In particular, members of the cytochrome P450 (CYP) CYP1, CYP2, and CYP3 families take part in the biotransformation of approximately 75% of all human drugs (Guengerich, 2008). These catalysts also perform cellular and systemic functions (e.g., growth, differentiation, apoptosis, homeostasis and neuroendocrine regulation) thanks to their ability to recognize a wide range of endogenous substrates. Following exposure to xenobiotics, CYP induction and/or inactivation could affect the therapeutic efficacy and/or toxicity of co-administered drugs (pharmacokinetic drug-food interactions). In particular, very little is known about the ability of geraniol to modulate the xenobiotic metabolizing enzymes.

Despite the potential therapeutic properties geraniol may exert in the body after oral administration, no pharmacokinetic and bioavailability data or information are currently available on the ability of this essential oil to cross the blood brain barrier. For this reason we performed a systematic high performance liquid chromatography (HPLC) study to identify the permeation properties of geraniol across intestinal cells and its pharmacokinetics and bioavailability after intravenous and oral administration to rats. As oral formulations we evaluated both “free” geraniol emulsified in glycerol to optimize its absorption in the bloodstream, and geraniol adsorbed in natural fibers to evaluate its ability to reduce systemic adsorption, thus allowing a potential local action of this essential oil in the gut. Taking into account the neuroprotective effects of geraniol (Rekha et al., 2013, 2014), we analyzed its concentration profile over time in the central nervous system of rat after oral administration of the “free” formulation. We also undertook a systematic investigation on hepatic phase I, phase II and antioxidant enzymatic activities and an in-depth hematochemical analysis in mice after oral administration of a high dose of geraniol (120 mg/kg body weight) for 4 weeks to verify its possible hepatotoxic effect.

## Materials and Methods

### Chemicals

Geraniol (98%) (PubChem CID: 637566), Carbazole (PubChem CID: 6854), HPLC-grade methanol, acetonitrile, ethyl acetate and water were acquired from Sigma–Aldrich. Fetal bovine serum (FBS), Dulbecco’s modified Eagle’s medium (DMEM) + Glutamax, streptomycin, penicillin, and phosphate-buffered saline (PBS) were obtained from Invitrogen (Life Technologies Italia, Milan, Italy). Geraniol adsorbed on natural fibers (BIOintestil^®^) was prepared following a patented procedure (Italian patent n° 102015000018215) and provided by Targeting Gut Disease Srl (Bologna, Italy).

Acetonitrile (PubChem CID:6342), aminopyrine (PubChem CID:6009), bovine serum albumin, dichlorophenolindophenol (PubChem CID:13726) (DCPIP), epinephrine (PubChem CID:5816), ethoxycoumarin (PubChem CID:35703), Folin-Ciocalteu reagent were from Sigma–Aldrich, glucose 6-phosphate (PubChem CID:5958) and glucose 6-phosphate dehydrogenase were from Roche Diagnostic (Indianapolis, IN, United States), L-glutathione oxidized (PubChem CID:71308714), L-glutathione reduced (PubChem CID:745), and methanol (PubChem CID:5958) were HPLC grade, methoxyresorufin (PubChem CID:119220), nicotinamide adenine dinucleotide phosphate in oxidized (PubChem CID:5886) and reduced forms (PubChem CID:5886) (NADP^+^ and NADPH), *p*-nitrophenol (PubChem CID:980), pentoxyresorufin (PubChem CID:107683), phenylmethylsulfonyl fluoride, resorufin (PubChem CID:69462), sodium dithionite (PubChem CID:24489), Triton X-100, 1-chloro-2,4-dinitrobenzene (PubChem CID:6), 1-naphthol, and 7-ethoxyresorufin (PubChem CID:3294) were from Sigma–Aldrich. All other chemicals were highest purity and are commercially available.

### HPLC Analysis

**FIGURE 2:**
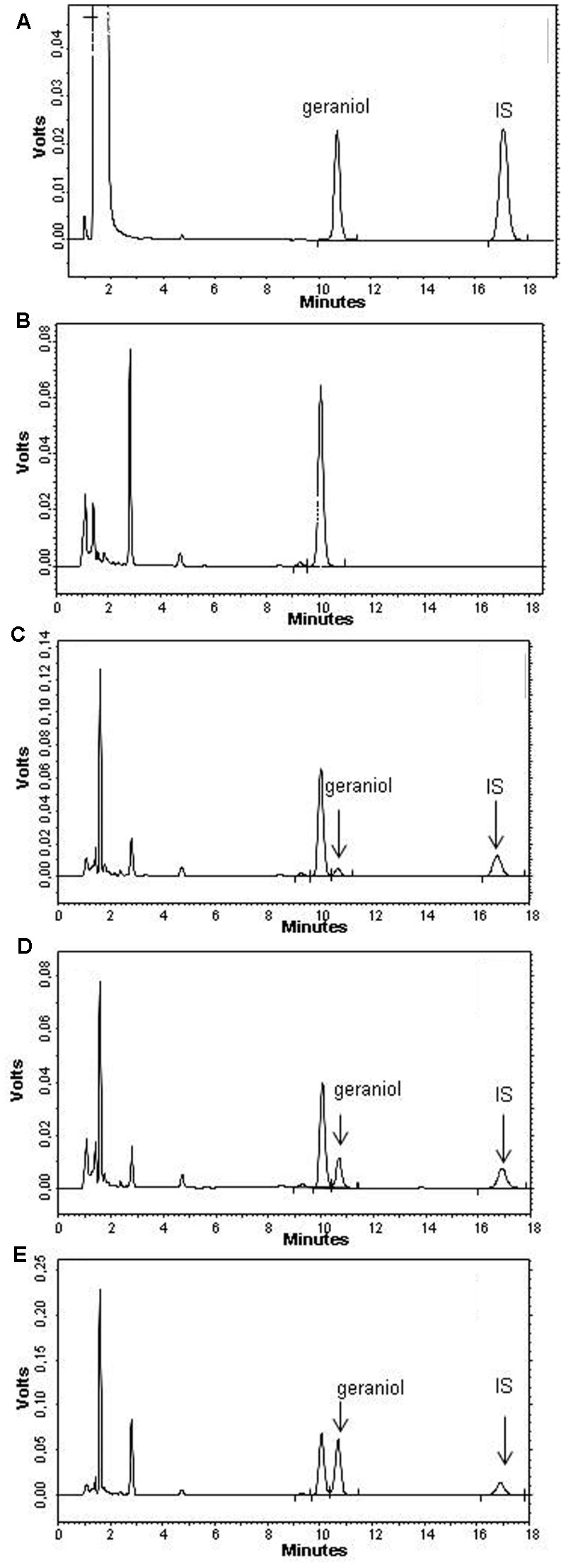
High performance liquid chromatography (HPLC) chromatograms for **(A)** a physical mixture of 6.4 μg/ml geraniol and 8.4 μg/ml carbazole used as internal standard (IS), **(B)** a blank blood sample and **(C–E)** geraniol extracted from blood samples at the concentrations 3.9 μg/ml **(C)**, 15.4 μg/ml **(D)** and 38.6 μg/ml **(E)**.

Geraniol was quantified by HPLC. The chromatographic apparatus consisted of a modular system (model LC-10 AD VD pump and model SPD-10A VP variable wavelength UV-Vis detector; Shimadzu, Kyoto, Japan) and an injection valve with a 20 μL sample loop (model 7725; Rheodyne, IDEX, Torrance, CA, United States). Separations were performed at room temperature on a 5-μm Hypersil BDS C-18 column (150 mm × 4.6 mm i.d.; Alltech Italia Srl, Milan, Italy) equipped with a guard column packed with the same Hypersil material. Data acquisition and processing were performed on a personal computer using CLASS-VP Software, version 7.2.1 (Shimadzu Italia, Milan, Italy). The detector was set at 210 nm; the mobile phase consisted of an isocratic mixture of water and acetonitrile at a ratio of 60:40 (v/v). The retention times obtained were 10.5 min for geraniol and 16.0 min for carbazole (**Figure 2A**), used as internal standard for the quantification of geraniol in blood samples (see below).

The chromatographic precision, represented by relative standard deviations (RSD), was evaluated by repeated analysis (*n* = 6) of the same sample water solution containing geraniol at a concentration of 20 μM (3.08 μg/mL). The RSD value was 0.9%. Geraniol was quantified by the peak area correlated with the predetermined standard curve over the range 1–200 μM (0.154–30.8 μg/mL). The calibration curve was linear (*n* = 9, *r* = 0.997, *P* < 0.001).

For cerebrospinal fluid (CSF) simulation, standard aliquots of balanced solution (PBS Dulbecco’s without calcium and magnesium) in the presence of 0.45 mg/mL BSA were employed (Felgenhauer, 1974; Madu et al., 1994). For the geraniol assay in CSF, the chromatographic precision was evaluated by repeated analysis (*n* = 6) of the same sample solution containing 5 μg/mL geraniol (RSD = 0.98%) in simulated CSF and calibration curves of peak areas versus concentration were generated in the range 0.1–10 μg/mL (*n* = 6, *r* = 0.997, *P* < 0.001).

Recovery experiments of 80 μg/mL geraniol from rat whole blood were performed by comparison of the peak areas extracted from blood test samples (see below) at 4°C (*n* = 6) with those obtained by injection of an equivalent concentration of analyte dissolved in its mobile phase. The average recovery ± SD was 62.4 ± 2.6%. The concentrations of this compound were therefore referred to as peak area ratio with respect to the internal standard carbazole. The precision of the method, evaluated by replicate analyses (*n* = 6) of rat blood extract containing the internal standard (carbazole) and geraniol at a level of 15 μg/mL, was demonstrated by the RSD value of 1.12%. Calibration standards were prepared by spiking blood extracts with the internal standard (carbazole) and with known amounts of geraniol corresponding to blood concentrations in the range 0.04–80 μg/mL at 4°C. These solutions were analyzed by HPLC and the calibration curve of peak area ratios versus concentrations was linear (*n* = 10, *r* = 0.996, *P* < 0.001).

Cerebrospinal fluid and blood matrix interferences on the HPLC chromatograms were also checked and were found not to interfere with the peak areas of geraniol and carbazole. **Figure 2B** reports a representative HPLC chromatogram for a blank blood sample, while **Figures 2C–E** show the HPLC chromatogram of geraniol ranging from 3.9 to 38.6 μg/ml extracted from blood samples.

### Stability Studies of Geraniol in Phosphate Buffered Saline

Geraniol was incubated at 37°C in 3 mL PBS, resulting in a final concentration of 100 μM (15.4 μg/ml) obtained by adding 1 μL of 10^-1^ M stock solution in DMSO for each milliliter incubated. During the experiment drug solutions were shaken continuously and gently in an oscillating water-bath. At regular time intervals 150 μL of samples were withdrawn and filtered (0.45 μm), then 10 μL were injected in the HPLC system for geraniol quantification.

### Cell Culture and Reagents

The NCM460 cell is an immortalized, non-transformed cell line derived from primary cells of the human transverse colonic mucosa (Moyer et al., 1996). This cell line was grown in DMEM + Glutamax supplemented with 10% FBS, 100 U/mL penicillin, and 100 μg/mL streptomycin at 37°C in a humidified atmosphere of 95%, with 5% CO_2_. For maximum viability, NCM460 cells were subcultured in fresh and spent growth medium at 1:1 ratio. All cell culture reagents were provided by Invitrogen (Life Technologies, Milan, Italy).

### Differentiation of NCM460 Cells to Polarized Monolayers

Differentiation to NCM460 cell monolayers was performed essentially according to the method reported by Ferretti et al. (2015). Briefly, after two passages, confluent NCM460 cells were seeded at a density of 10^5^ cells/mL in a 1:1 ratio of fresh and spent culture medium in 12-well Millicell inserts (Millipore, Milan, Italy) consisting of 1.0 μm pore size polyethylene terephthalate (PET) filter membranes, whose surface was 1.12 cm^2^. Filters were presoaked for 24 h with fresh culture medium, and then the upper compartment (apical, A) received 400 μL of the diluted cells, whereas the lower compartment (basolateral, B) received 2 mL of the medium in the absence of cells. Half volume of the culture medium was replaced every 2 days with fresh medium to each of the apical and basolateral compartments. The integrity of the cell monolayers was monitored by measuring the transepithelial electrical resistance (TEER) by means of a voltmeter (Millicell-ERS; Millipore, Milan, Italy). The measured resistance value was multiplied by the area of the filter to obtain an absolute value of TEER, expressed as Ω⋅cm^2^. The background resistance of blank inserts not plated with cells was around 35 Ω⋅cm^2^ and was deducted from each value. The homogeneity and integrity of the cell monolayer were also monitored by phase contrast microscopy. Based on these parameters, cell monolayers reached confluence and epithelial polarization after 6 days, and monolayers with a stable TEER value around 180 Ω⋅cm^2^ were used for permeation studies. At this point, the medium was replaced with low serum fresh medium (1% FBS) in both the apical and basolateral compartments and left for 24 h.

### Permeation Studies across Cell Monolayers

Permeation experiments were performed in triplicate in both the apical-to-basolateral (A → B) and basolateral-to-apical (B → A) directions. To this end, a solution of geraniol was separately prepared in PBS containing 5 mM glucose at a concentration of 500 μM, obtained by adding 5 μL of 1⋅10^-1^ M stock solution in DMSO for each milliliter incubated. The fresh low-serum media-cultured monolayers of FHC cells were removed from both the A and B sides of the inserts, and both sides were washed twice with pre-warmed PBS. During transport experiments, the Millicell systems were continuously swirled on an orbital shaker (100 rpm) at 37°C. For the A → B permeation studies, 0.4 mL of geraniol solution was added to the apical side at time t = 0, and the inserts were placed in a cell culture plate whose basolateral compartment was pre-filled with 2 mL of pre-warmed PBS containing 5 mM glucose. At the pre-established time points, the inserts were removed and transferred to a new well-plate containing fresh PBS with glucose. The contents of the basolateral compartment were collected after insert removal, and 10 μL aliquots of filtered (0.45 μm) samples were immediately injected into the HPLC apparatus. For the B → A permeation studies, at time *t* = 0, 2 mL of geraniol solution were placed on the basolateral side of Millicell inserts whose apical side contained 0.4 mL of fresh PBS with glucose. At the predetermined time points, the apical samples were removed and replaced with fresh PBS containing glucose. The collected apical samples were immediately filtered (0.45 μm) and injected (10 μl) into the HPLC apparatus. TEER values were monitored before and after each experiment. Permeation studies were also conducted using cell-free inserts under the same conditions described above as control. All values obtained were the means of four independent experiments.

Apparent permeability coefficients (P_app_) of the analyzed compounds were calculated according to the following equation (Artursson and Karlson, 1991; Pal et al., 2000; Raje et al., 2003):

$${\text{P}}_{\text{app}}=\frac{\frac{\text{dc}}{\text{dt}}{\text{V}}_{\text{r}}}{{\text{S}}_{\text{A}}{\text{C}}_{0}}$$

where P_app_ is the apparent permeability coefficient in cm/min; dc/dt (μM/min) is the flux of drug across the filters, calculated as the linearly regressed slope of accumulated concentrations (μM) in the receiving compartment; V_r_ is the volume in the receiving compartment (Apical = 0.4 mL; Basolateral = 2 mL); S_A_ is the diffusion area (1.13 cm^2^); and C_0_ is the initial compound concentration in the donor chamber (μM) at *t* = 0. The permeabilities were determined for the filters alone (P_f_), and for the filters covered with cells (P_t_). The apparent permeability coefficients (P_E_) of the cell monolayer were then calculated as follows (Yee, 1997; Raje et al., 2003):

$$\frac{\text{1}}{{\text{P}}_{\text{E}}}\text{=}\frac{\text{1}}{{\text{P}}_{\text{t}}}\text{-}\frac{\text{1}}{{\text{P}}_{\text{f}}}$$

### Stability Studies of Geraniol in Human or Rat Whole Blood

Geraniol was incubated at 37°C in 3 mL of human or rat whole blood, resulting in a final concentration of 167 μM (25.7 μg/ml) obtained by adding 5 μL of 10^-1^ M stock solution in DMSO to the blood. Human blood was obtained from healthy volunteers. Rat blood was obtained from male Sprague-Dawley rats (Charles-River, Milan, Italy). During the experiment the samples were shaken continuously and gently in an oscillating water-bath. At regular time intervals 100 μL of samples were withdrawn, immediately hemolyzed in Eppendorf tubes prefilled with 500 ml of water (HPLC grade, about 4°C), then 50 μL of 3 M sodium hydroxide and 50 μL of internal standard solution (50 μM carbazole) were added. The samples were extracted twice with 1 ml of water-saturated ethyl acetate. After centrifugation (10 min at 13,000 × *g*), the organic layer was reduced to dryness by N_2_ flow, then 150 μL of a H_2_O:CH_3_CN mixture (50:50 v/v) were added and, after centrifugation, 10 μl were injected into the HPLC system for geraniol and carbazole detection.

### *In Vivo* Geraniol Administration and Quantification

#### Intravenous Infusion

Male Sprague-Dawley rats (200–250 g) were anesthetized during the experimental period and received a femoral intravenous infusion of 12.5 mg/mL of geraniol dispersed in a medium constituted by 20% (v/v) DMSO and 80% (v/v) physiologic solution in the presence of 0.82 mg/mL sodium taurocholate, at a rate of 0.2 mL/min for 5 min. At the end of the infusion and at fixed time points, blood samples (100 μL) were collected and immediately hemolyzed with 500 μL of ice cold water, then 50 μL of 3M sodium hydroxide and 50 μL of internal standard solution (50 μM carbazole) were added. The samples were extracted and analyzed as described above, with the only difference that the samples withdrawn within 20 min after infusion were diluted 1:10 using the H_2_O:CH_3_CN mixture (50:50 v/v) before analysis. Four rats ware employed for intravenous infusions of geraniol.

The *in vivo* half-life of geraniol in the rat bloodstream was calculated by non-linear regression (exponential decay) of concentration values in the time range within 3 h after infusion and confirmed by linear regression of the log concentration values versus time. The calculations were performed using the computer program GraphPad Prism.

#### Oral Administration

Adult male Sprague-Dawley rats (200–250 g body weight) fasted for 24 h received an oral gavage dose of 50 mg/kg geraniol (about 12.5 mg) in two types of formulations: in the first formulation (immediate release) geraniol was emulsified in 1 mL of anhydrous glycerol; in the second formulation (delayed release) geraniol was contained in vegetable fiber (BIOintestil^®^). At the end of the oral administration and at fixed time points, blood samples (100 μL) were collected and CSF samples (50 μL) were withdrawn using the cysternal puncture method described by van den Berg et al. (2002), which requires a single needle stick and allows the collection of serial (40–50 μL) CSF samples that are virtually blood-free (Dalpiaz et al., 2014). A total volume of approximately 150 μL of CSF was collected during the experimental session. The CSF samples (10 μL) were immediately injected into the HPLC system for geraniol detection. The blood samples were extracted and analyzed as described above; the samples withdrawn within 40 min after infusion were diluted 1:10 using the H_2_O:CH_3_CN mixture (50:50 v/v) before analysis The blood or CSF geraniol concentrations at the programmed time points were detected in at least four rats following each type of oral administration. In particular, blood withdrawals for all programmed time points were repeated four times with a corresponding number of rats; CSF withdrawals for all programmed time points were repeated at least four times with a number of rats allowing no more than three CSF withdrawals from each of them. The area under the concentration curves of geraniol in the rat bloodstream (AUC, μg mL^-1^ min) following intravenous infusion and oral administrations were calculated using the trapezoidal method. The absolute bioavailability values of geraniol, referred to the oral administered formulations, were obtained as the ratio between their oral AUC values and the AUC of the intravenous administration of the drug. All the calculations were performed using the computer program Graph Pad Prism.

#### Hepatic Enzymatic Perturbations

Sixteen 8-week-old male C57BL/6 mice were purchased from Charles River Laboratories (Lecco, Italy). Animals were housed in collective cages with a controlled environment containing two mice each, at 22 ± 2°C and 50% humidity, under a 12-h light/dark cycle. Mice were allowed to acclimate to these conditions for at least 7 days before inclusion in experiments and had free access to food and water throughout the study. Mice were randomized into two experimental groups: the first (I) group (CTRL) (*n* = 10) was treated with the vehicle (glycerol), the second (II) group (*n* = 10) with geraniol free suspension at 120 mg kg^(-1)^ body weight, both by gavage for 28 consecutive days. At the end of treatment, mice were killed. Blood (2 vial containing 0.5 ml each) was collected for hematochemical analysis and lipid serum profile. Livers were collected to evaluate enzymatic activities on liver microsomal fraction.

#### Animal Care and Treatment

The experimental procedures were carried out in conformity with protocols endorsed by the National Academy of Science guidelines and in accordance with EU Directive 2010/63/EU on the protection of animals used for scientific purposes. All efforts were made to minimize animal suffering. The experiments using rats were approved by the Committee on the Ethics of Animal Experiments of the University of Ferrara while those on mice were approved by the Committee on the Ethics of Animal Experiments of the University of Bologna. Both experimental protocols were approved by the Italian Ministry of Health.

### Tissue Collection and Preparation of Subcellular Fractions

The tissue collection and isolation of hepatic subcellular fractions were previously reported (Canistro et al., 2017). Livers were weighed and thereafter immediately removed from each rat and processed separately. The S9 fraction and the post-mitochondrial supernatant were prepared as described by Bauer et al. (1994). The fractions were immediately frozen in liquid nitrogen, stored at -80°C and used within a week for enzymatic analyses. In this short period, the activities measured in conditions of V_max_ and linearity of protein contents were stable.

### Hepatic Phase I, Phase II and Antioxidant Enzymes

The methods for phase I, phase II enzyme assays were recently described (Canistro et al., 2016). Antioxidant enzyme activities were measured as defined by Melega et al. (2013). All details relating to the procedure are reported here.

#### Phase I Enzymatic Activities

NADPH-(CYP)-c-reductase (CYP-red) activity. The analytical method is based on the determination of the reduction rate of cytochrome c at 550 nm (𝜀 = 19.1 mM^-1^ cm^-1^), according to previously defined procedures (Bruce, 1967; Canistro et al., 2012b). The incubation mixture contained 1.6 ml of 0.05 M Tris-HCl buffer (pH = 7.7) with 0.1 mM EDTA + 0.5 mg cytochrome c + 0.2 ml of microsomes. The reaction begins with the addition of 0.2 ml NADPH. Specific reaction was read at 550 nm against buffer plus cytochrome c.

Aminopyrine *N*-demethylase (APND) activity-CYP3A1/2. Activity was determined by the quantification of CH_2_O release, according to Mazel (1971). The total incubation volume was 3 ml, composed of 0.5 ml water solution of 50 mM aminopyrine and 25 mM MgCl_2_, 1.48 ml of 0.60 mM NADP^+^, 3.33 mM G6P in 50 mM Tris-HCl buffer (pH 7.4), 0.02 ml G6PDH (0.93 U/ml) and 0.125 ml of sample. After 5 min of incubation at 37°C, the yellow color developed by the reaction of the released of CH_2_O with the Nash reagent was read at 412 nm, and the molar absorptivity of 8,000 used for calculation (Melega et al., 2013).

*p*-nitrophenol hydroxylase (p-NPH) activity-CYP2E1. Activity was determined in a final volume of 2 ml: 2 mM *p*-nitrophenol in 50 mM Tris-HCl buffer (pH 7.4), 5 mM MgCl_2_, and a NADPH-generating system consisting of 0.4 mM NADP^+^, 30 mM isocytrate, 0.2 U of isocytrate dehydrogenase and 1.5 mg of proteins. After 10 min of incubation at 37°C, the reaction was terminated by addition of 0.5 ml of 0.6 N perchloric acid. Precipitated proteins were removed by centrifugation and 1 ml of the resultant supernatant was mixed with 1 ml of 10 N NaOH. Absorbance at 546 nm was immediately recorded and 4-nitrocathecol determined (𝜀 = 10.28 mM^-1^ cm^-1^) (Canistro et al., 2012a).

Pentoxyresorufin *O*-dealkylase (PROD) activity-CYP2B1/2, ethoxyresorufin *O*-deethylase, (EROD)-CYP1A1 and methoxyresorufin *O*-demethylase (MROD)-CYP1A2. Reaction mixture (PROD) consisted of 0.025 mM MgCl_2_, 200 mM pentoxyresorufin, 0.32 mg of proteins and 130 mM NADPH in 2.0 ml 0.05 M Tris-HCl buffer (pH 7.4). Resorufin formation at 37°C was calculated by comparing the rate of increase in relative fluorescence to the fluorescence of known amounts of resorufin (excitation 563 nm, emission 586 nm) (Lubet et al., 1985). EROD and MROD activities were measured in exactly the same manner as described for the pentoxyresorufin assay, except that substrate concentration was 1.7 mM for ethoxyresorufin and 5 mM for methoxyresorufin (Sapone et al., 2016).

Ethoxycoumarin *O*-deethylase (ECOD) activity-CYP1A1/2, CYP2A, CYP2B, CYP2E1. ECOD was determined by the quantification of umbelliferone formation, according to Aitio (1978). Incubation mixture consisted of 2.6 ml, composed of 1 mM ethoxycoumarin, 5 mM MgCl_2_, NADPH-generating system (see aminopyrine assay) and 0.25 ml of sample. After 5 min of incubation at 37°C, the reaction was stopped by the addition of 0.85 ml of trichloroacetic acid (TCA) 0.31 M. The pH of the mixture was brought to about 10 by adding 0.65 ml of 1.6 M NaOH-glycine buffer (pH 10.3); the amount of umbelliferone was measured fluorimetrically (excitation 390 nm; emission 440 nm) (Vivarelli et al., 2016b).

#### Phase II Enzymatic Activities

##### Glutathione *S*-transferase (GST) activity

The incubation mixture for measuring overall GST activity contained 1 mM glutathione + 1 mM 1-chloro-2,4-dinitrobenzene (CDNB) in methanol + 0.025 mL of sample in a final volume of 2.5 mL 0.1 M phosphate Na^+^/K^+^ buffer (pH 6.5). The product of the reaction of the thiol group of glutathione with the electrophilic group of CDNB was read at 340 nm (𝜀 = 9.6 mM^-1^ cm^-1^) (Vivarelli et al., 2016a).

##### UDP-glucuronosyl transferase (UDPGT) activity

The overall UDPGT activity was determined kinetically using 1-naphthol as substrate (final concentration, 50 mM) by the continuous fluorimetric (excitation 390 nm; emission 440 nm) monitoring of 1-naphtholglucuronide production in the presence of 1 mM uridine-5′-diphosphoglucuronic acid (Mackenzie and Hänninen, 1980). Experiments were performed in the presence or absence of Triton X-100 (0.2%) as a detergent, in order to improve the assay sensitivity (Canistro et al., 2008).

#### Antioxidant Enzymatic Activities

Catalase (CAT) activity. The reaction was started in a quartz cuvette containing 50 mM potassium phosphate buffer and cytosol sample by adding 30 mM H_2_O_2_. The decomposition of the substrate was measured at 240 nm and catalase activity was expressed as mol of H_2_O_2_ consumed per minute per mg protein using a molar extinction coefficient of 43.6 mM^-1^ cm^-1^ (Bonamassa et al., 2016).

NAD(P)H:quinone reductase (NQO1) activity. NQO1 activity was assayed spectrophotometrically at 600 nm by monitoring the reduction of the blue redox dye of DCPIP (𝜀 = 9.6 mM^-1^ cm^-1^), and expressed as mol of DCPIP reduced per minute per mg protein (Bonamassa et al., 2016).

Oxidized glutathione reductase activity (GSSG-red). GSSG-red activity was measured by adding 1.5 mM NADPH to an assay cuvette containing 50 mM potassium phosphate buffer, 1 mM EDTA, cytosol sample and 20mM GSSG. The generation of NADP^+^ from NADPH during the reduction of GSSG was recorded at 340 nm for 5 min at 37°C. GSSG-red activity was calculated using the extinction coefficient of 6.22 per mM x cm, and expressed as mol of NADPH consumed/min per mg protein (Bonamassa et al., 2016).

Superoxide dismutase activity (SOD): Superoxide dismutase activity activity was determined according to Misra and Fridovich’s assay (Misra and Fridovich, 1972). Briefly, the activity was assayed spectophotometrically at 320 nm by monitoring the generation of adenochrome, one of the main products of epinephrine autoxidation at pH 10.2. The dejection of autoxidation was used to calculate SOD activity using the extinction coefficient of 4.02 per mM × cm, and expressed as mol of epinephrine oxidized/min per mg protein, derived by subtracting each test curve from the epinephrine autoxidation standard curve (Bonamassa et al., 2016).

#### Hematochemical Analysis

For each animal, blood samples were collected in both heparinized and non-heparinized tubes. Plasma from heparinized samples was obtained after tabletop centrifugation at 2,000 rpm, while blood in non-heparinized tubes was centrifuged at 1,500 rpm for 10 min after complete coagulation, to obtain serum. Biochemical and hematological analyses were assessed by the Department of Veterinary Medical Science, University of Bologna.

#### Protein Concentration

Protein concentration was determined according to the Lowry method revised in Bailey (1967) using bovine serum albumin as standard and diluting samples 200 times to provide a suitable protein concentration.

### Statistical Analysis

Statistical comparisons of permeability coefficients obtained from the transport studies were made by Student’s *t*-test (GraphPad Prism, GraphPad Software, Incorporated, La Jolla, CA, United States). *P* < 0.05 was considered statistically significant. GraphPad Prism was employed for linear regression of the cumulative amounts of the compounds in the receiving compartments of the Millicell systems. The quality of fit was determined by evaluating the correlation coefficients (r) and P values. Statistical comparisons of AUC values and hematochemical data were made by one way ANOVA followed by Bonferroni test (GraphPad Prism). *P* < 0.05 was considered statistically significant. Results from xenobiotic metabolizing and antioxidant enzymes were analyzed using the two-tailed unpaired *t*-test with the multiple-comparison *post hoc* analysis (Holm-Sedak).

## Results

### Permeation Studies across Cell Monolayers

The first step of our work consisted of permeability studies evaluating the bidirectional transport of geraniol across an *in vitro* model of human intestinal wall, i.e., NCM460 cell monolayers. Glucose-enriched PBS was used as an incubation medium for the permeability studies. As a consequence, before starting these studies we verified that geraniol dissolved in PBS is highly stable at 37°C for at least 8 h (data not shown).

**FIGURE 3:**
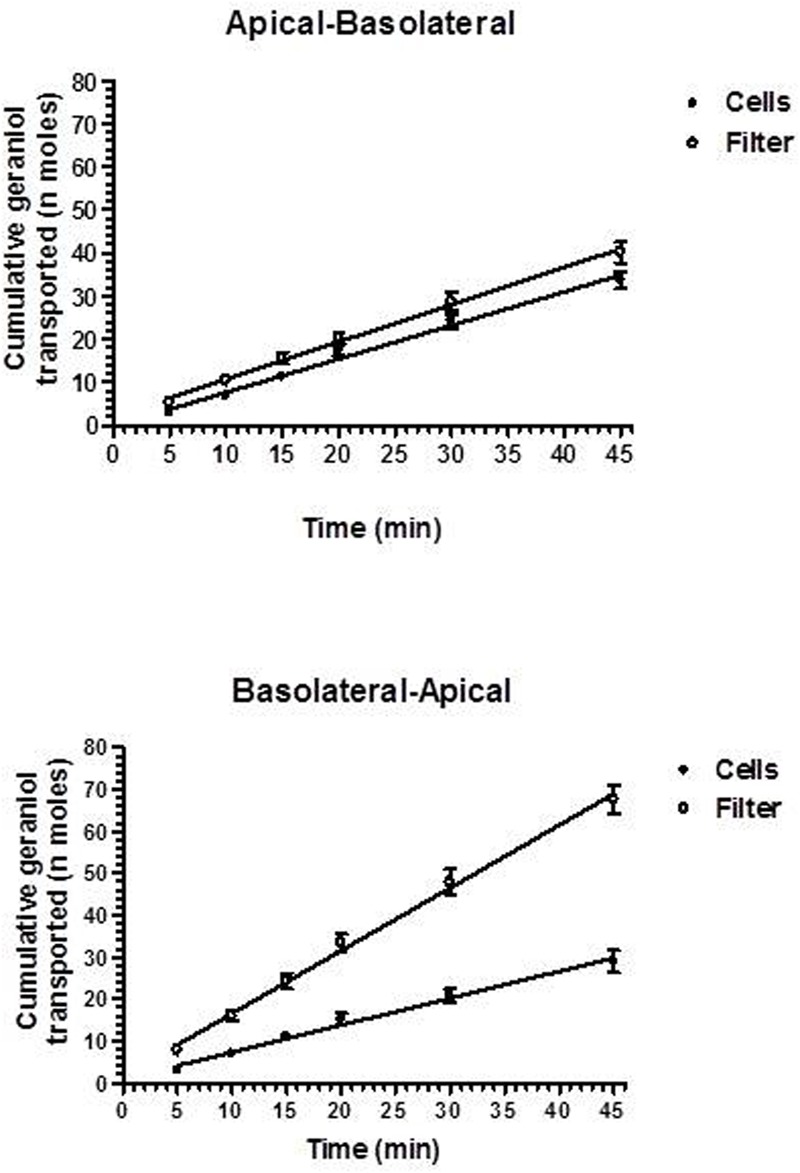
Permeation kinetics of 500 μM geraniol across the Millicell filters alone (filter) or covered by monolayers obtained from NCM460 cells. The permeations were analyzed from the apical to basolateral compartments and from the basolateral to apical compartments. In all cases analyzed, the cumulative amounts in the receiving compartments were linear over time within 45 min (*r* ≥ 0.994, *P* ≤ 0.0001). All data are reported as mean ± SE of four independent experiments.

The permeability studies were performed in both the apical-to-basolateral (A → B) and basolateral-to-apical (B → A) directions after cell cultures reached the confluence on parallel sets of Millicell well plates with similar TEER values (180 ± 11 Ω⋅cm^2^). The permeation profiles referred to both directions of geraniol across the Millicell filters alone (Filter) or covered with monolayers of NCM460 cells are reported in **Figure 3** showing that the cumulative amounts of geraniol (nanomoles) in the receiving compartments have a linear profile over 45 min in all cases (*r* ≥ 0.994, *P* ≤ 0.0001), indicating constant permeation conditions within this range of time. The slopes of the linear fits, referred to accumulated concentrations (μM) in the receiving compartments, were used according to equation 1 to calculate the permeability coefficients (P_t_ and P_f_), which were in turn used as per equation 2 to calculate the apparent permeability coefficients (P_E_) of geraniol specific for the cell monolayers, whose values for A → B and B → A transport of geraniol were 13.10 ± 2.3 ⋅ 10^-3^ and 2.1 ± 0.1 ⋅ 10^-3^ cm/min, respectively, indicating that the permeation rate of this drug from the apical to the basolateral compartments was roughly sixfold higher than its permeation rate in the opposite direction (*P* < 0.001).

### Geraniol Pharmacokinetics

The second step of our work was the evaluation of the kinetic elimination of geraniol from the bloodstream and its oral bioavailability after administration to rats. As a consequence, the same dose of intravenous and oral geraniol (50 mg/kg) was administered. Before starting the *in vivo* administrations of geraniol, we evaluated the drug’s stability *in vitro* at 37°C in human and rat whole blood. Our results indicated that geraniol is highly stable in these systems within 8 h (data not shown), showing that the whole blood of human and rodents is not able to induce geraniol degradation.

**FIGURE 4:**
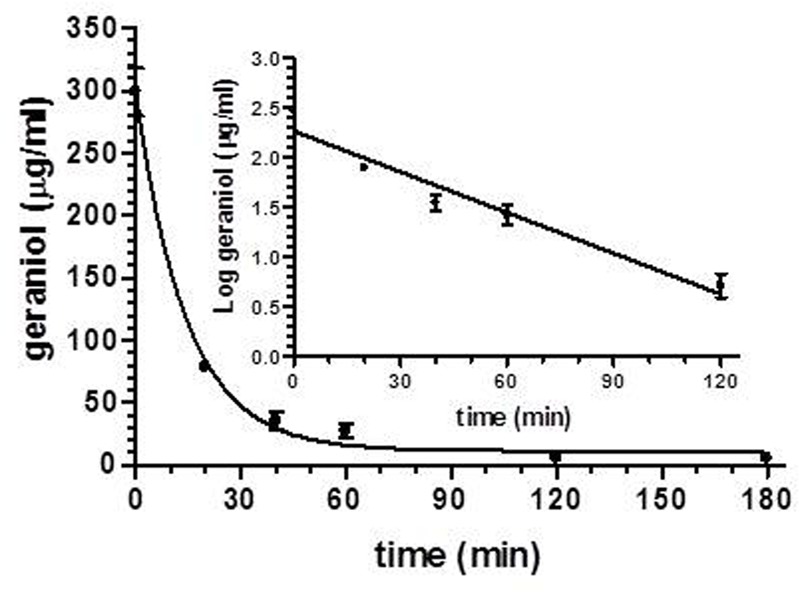
Elimination profile of geraniol after 12.5 mg intravenous infusion to rats. Data are expressed as the mean ± SE of four independent experiments. The elimination followed an apparent first order kinetics, confirmed by the semilogarithmic plot reported in the inset (*n* = 5, *r* = 0.963, *P* < 0.0001). The half-life of geraniol was calculated to be 12.5 ± 1.5 min.

After intravenous infusion of 12.5 mg of geraniol, the drug concentration in the rat bloodstream was 298 ± 19 μg/mL. This value decreased over time with an apparent first order kinetics (**Figure 4**), confirmed by the linearity of the semilogarithmic plot reported in the inset of **Figure 4** (*n* = 5, *r* = 0.963, *P* < 0.0001), showing a half-life of 12.5 ± 1.5 min.

**FIGURE 5:**
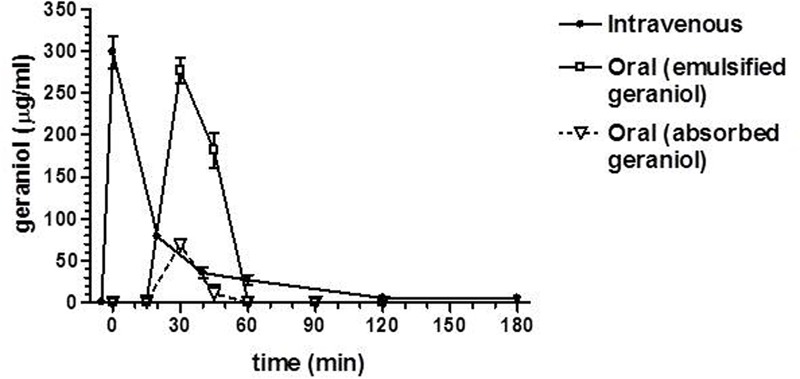
Blood geraniol concentrations (μg/mL) within 180 min after intravenous infusion (iv) or oral administration of a 12.5 mg dose to rats. Data are expressed as the mean ± SE of four independent experiments. The oral formulations consisted of emulsified geraniol or a suspension of geraniol adsorbed in vegetable fiber.

**FIGURE 6:**
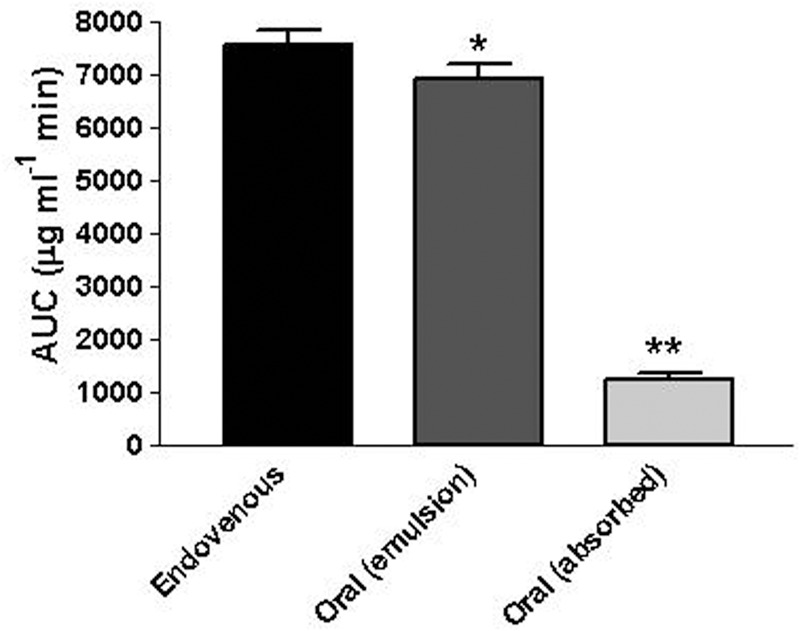
Area Under Concentration values (AUC) referred to the bloodstream of rats obtained after intravenous infusion or oral administration of a 12.5 mg dose of geraniol. Data are expressed as the mean ± SE of four independent experiments. ^∗^*P* > 0.05 versus intravenous; ^∗∗^*P* < 0.001 versus intravenous.

**Figure 5** reports the rat blood geraniol concentrations within 3 h after its intravenous infusion or the oral administration of 50 mg/Kg dose as geraniol emulsified in glycerol or as geraniol suspension adsorbed on vegetable fiber. These two oral formulations induced maximum concentration peaks (C_max_) in the rat bloodstream 30 min after administration, showing different values. In particular, the C_max_ value of the emulsified formulation was relatively high (276 ± 15 μg/ml) being similar to the geraniol concentration detected at the end of intravenous infusion. Conversely, the C_max_ value of the fiber-adsorbed formulation was strongly reduced (69 ± 4 μg/ml), appearing fourfold lower than the C_max_ of the oral emulsified formulation. These data indicate different modalities of geraniol adsorption in the bloodstream of rats from the oral administrations, as confirmed by the AUC values reported in **Figure 6**. In particular, as hypothesized by the permeation studies, geraniol in the free form (emulsified formulation) was easily absorbed in the bloodstream, yielding an AUC value of 6911 ± 279 μg⋅mL^-1^⋅min, not significantly dissimilar (*P* > 0.05) to the AUC value of the intravenous administration (7561 ± 300 μg⋅mL^-1^⋅min). On the other hand, the geraniol absorbed on vegetable fiber showed a drastically reduced AUC value (1233 ± 114 μg⋅mL^-1^⋅min), about fivefold lower than the AUC values associated with the oral emulsified formulation (*P* < 0.001). According to the AUC values reported in **Figure 6**, the absolute bioavailability values of the oral formulations of emulsified geraniol (immediate release) or fiber-adsorbed geraniol (delayed-release) were 92 and 16%, respectively. The fiber-association process strongly reduces geraniol bioavailability, much more than the geraniol encapsulation in soy lecithin micelles adopted by De Fazio et al. (2016) that presented an absolute bioavailability of 50% (data not shown).

**FIGURE 7:**
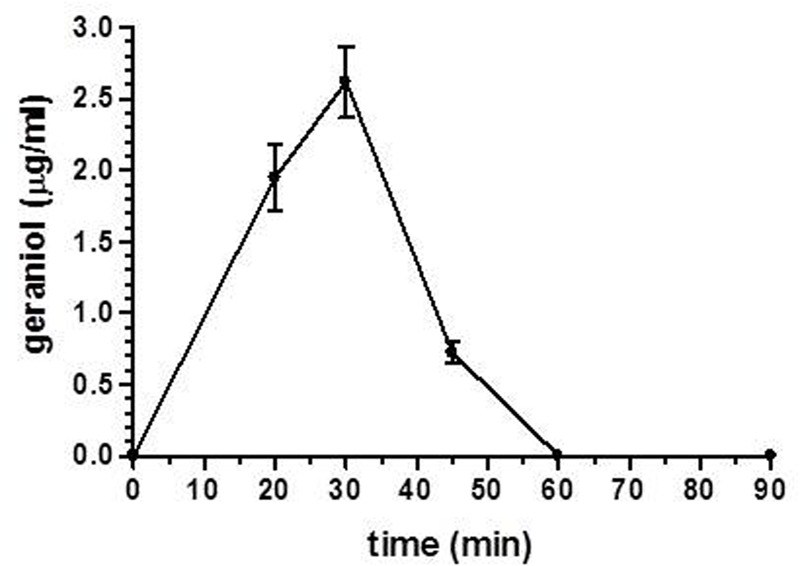
Geraniol concentrations (μg/mL) detected in the CSF of rats after oral administration of a 12.5 mg dose as emulsified formulation. Data are expressed as the mean ± SE of at least four independent experiments.

Finally, following the oral administration of the emulsified oral formulation, we evaluated the geraniol amounts in the CSF of rats. As reported in **Figure 7**, geraniol concentrations were quantified in the CSF of rats in a range between 0.72 ± 0.08 μg/mL and 2.6 ± 0.2 μg/mL within 60 min after administration. The maximum peak of concentration was reached 30 min after oral administration, as recorded for geraniol in the bloodstream. These results demonstrate geraniol’s ability to permeate the central nervous system from the bloodstream.

### Effects of Geraniol on Enzymatic Activities in Mice Liver

As reported in **Table 1**, each enzymatic activity tested by single-probe-based assays, except for the dealkylase of pentoxyresorufin and the deethylase of ethoxyresorufin, was found downregulated in mice by geraniol (*P* < 0.01). CYP (red) activity was decreased up to 18% (*P* < 0.01), CYP3A1/2 up to 54% (*P* < 0.01), CYP2E1 up to 23% (*P* < 0.01) and CYP1A2 up to 15% (*P* < 0.01) with respect to controls. Ethoxycoumarin *O*-deethylase activity was not influenced by geraniol. **Table 2** shows that Phase II enzymes were not affected by the treatment. As shown in **Table 3**, geraniol caused a significant increment of antioxidant activity for almost all enzymes tested. In particular, catalase (CAT) was induced up to 29% (*P* < 0.01), NADPH quinone reductase (NQO1) up to 211% (*P* < 0.01) and oxidized glutathione reductase (GSSG-red) up to 56% (*P* < 0.01) with respect to controls. Superoxide dismutase (SOD) activity was not affected by geraniol supplementation.

**Table 1:** Effects of geraniol on phase I enzymatic activities in mice liver.

	CTRL		Geraniol	
Parameters	Mean	*SD*	Mean	*SD*
NADPH-cytochrome (P450) reductase (nmol × mg^-1^ × min^-1^)	28.37	0.69	23.30	2.22**
Aminopyrine *N*-demethylase (nmol × mg^-1^ × min^-1^) (CYP3A1/2)	4.60	0.42	2.10	0.36**
*p*-nitrophenol hydroxylase (nmol × mg^-1^ × min^-1^) (CYP2E1)	0.39	0.01	0.30	0.01**
Ethoxycoumarin *O*-deethylase (nmol × mg^-1^ × min^-1^)	1.56	0.14	1.53	0.10
Pentoxyresorufin *O*-dealkylase (pmol × mg^-1^ × min^-1^) (CYP2B1/2)	9.32	0.86	11.30	0.51**
Ethoxyresorufin *O*-deethylase (pmol × mg^-1^× min^-1^) (CYP1A1)	22.12	1.29	25.50	1.05**
Methoxyresorufin *O*-demethylase (pmol × mg^-1^ × min^-1^) (CYP1A2)	17.16	0.45	14.57	1.03**

*Values are mean ± standard deviation (*SD*) of ten measurements performed on 10 mouse samples for each studied group. Mean values were significantly different from those of the CTRL group: ^∗^*P* < 0.05, ^∗∗^*P* < 0.01, two-tailed *t*-test.*

**Table 2:** Effects of geraniol on phase II enzymatic activities in mice liver.

	CTRL		Geraniol	
Parameters	Mean	*SD*	Mean	*SD*
Glutathione *S*-transferase (μmol × mg^-1^ × min^-1^) (GST)	2.26	0.17	2.60	0.27
UDP glucuronosyl-transferase (nmol × mg^-1^ × min^-1^) (UDPGT)	4.17	0.22	3.89	0.18

*Values are mean ± standard deviation (*SD*) of ten measurements performed on ten mouse samples for each studied group. Mean values were significantly different from those of the CTRL group: ^∗^*P* < 0.05, ^∗∗^*P* < 0.01, two-tailed *t*-test.*

**Table 3:** Effects of geraniol on antioxidant enzymatic activities in mice liver.

	CTRL		Geraniol	
Parameters	Mean	*SD*	Mean	*SD*
Catalase (μmol × mg^-1^ × min^-1^) (CAT)	1.98	0.04	2.55	0.11**
NAD(P)H: quinone reductase (nmol × mg^-1^ × min^-1^) (NQO1)	3.87	0.27	12.43	1.40**
Superoxide dismutase (nmol × mg^-1^ × min^-1^) (SOD)	12.72	1.36	13.65	0.81
Oxidized glutathione reductase (μmol × mg^-1^ × min^-1^) (GSSG-red)	75.73	10.09	118.31	8.76**

*Values are mean ± standard deviation (*SD*) of 10 measurements performed on ten rat samples for each studied group. Mean values were significantly different from those of the CTRL group: ^∗^*P* < 0.05, ^∗∗^*P* < 0.01, two-tailed *t*-test.*

### Effects of Geraniol on Liver Damage or Injury

As shown in **Table 4**, geraniol treatment did not affect ALT and AST blood concentrations or serum lipid values.

**Table 4:** Effect of geraniol administration on selected blood parameters.

	CTRL	Geraniol
AST (U/l)	157 ± 23	149 ± 24
ALT (U/l)	32 ± 6	34 ± 5
TG (mg/dl)	174 ± 15	163 ± 13
Cholesterol (mg/dl)	91 ± 5	94 ± 7
HDL cholesterol (mg/dl)	69 ± 4	66 ± 4

*Metabolic serum parameters and transaminase levels of C57BL76 mice treated for 28 days with a high dose of geraniol [free suspension, 120 mg kg^(-1)^ body weight] compared to untreated mice. Results are expressed as mean ± SEM (*n* = 10).*

## Discussion

Geraniol is a natural monoterpene classified in the GRAS category (Lapczynski et al., 2008). Its potential therapeutics include anti-inflammatory, antioxidant, neuroprotective and anticancer effects, often evidenced following oral administration of doses ranging from 50 mg/Kg to more than 200 mg/Kg (Rekha et al., 2013, 2014; Cho et al., 2016; De Fazio et al., 2016). The potential clinical use of geraniol appears promising, so the knowledge of *in vivo* pharmacokinetics and bioavailability data are essential to design appropriate geraniol formulations and plan adequate therapeutic protocols. It is known that first phase metabolites of geraniol can be isolated from urine after oral administration to rodents (Chadha and Madyastha, 1984; Scheline, 1991), but to the best of our knowledge, no pharmacokinetic and bioavailability data are currently available. For this reason we undertook an experimental study to clarify *in vitro* the modalities that allow geraniol to permeate from the intestinal lumen to the bloodstream. We also evaluated *in vivo* pharmacokinetic and bioavailability data following intravenous and oral administrations of geraniol to rats, and the potential hepatotoxic effect of high dose geraniol administration (120 mg/kg) to mice.

We performed the *in vitro* permeability studies of geraniol across monolayers of NCM460 cells. Human normal colonic epithelial NCM460 cells represent a non-transformed and non-tumorigenic cell line derived from primary cells of the normal human transverse colon mucosal epithelium (Moyer et al., 1996). This model has proved useful in multiple intestinal research areas, including studies on the bioactivity of dietary compounds (Olejnik et al., 2016). After confluence in Millicell systems, these cells acquire functional polarization that allows them to form a monolayer and act as an epithelial barrier (Ferretti et al., 2015; Valerii et al., 2015) showing transepithelial electrical resistance (TEER) values closer to the range reported for intact sheets of human colonic mucosa than those developed by Caco-2 cells (Sahi et al., 1998; Liu et al., 2010). Therefore, NCM460 cells were chosen as an *in vitro* model system of a human intestinal barrier in our study. These cells retain more physiological characteristics of the normal human colon than other consolidated models, such as Caco-2 cells, where the loss of contact inhibition and polarization in transformed cells is known to produce changes in growth characteristics (monolayers/multilayers) (Rothen-Rutishauser et al., 2000).

After confluence in Millicell systems, the NCM460 cell layer separated into an upper and lower compartment. The upper compartment represented the apical side of the NCM460 cells, corresponding to the intestinal lumen facing the domain of the monolayer, whereas the lower compartment represented the basolateral side of the NCM460 cells, corresponding to the blood-facing side of the monolayer (Pereira et al., 2016). This system provided a very useful tool to simulate *in vitro* the permeation of geraniol across the intestinal barrier. In particular, the permeation properties of geraniol were shown by diffusion studies in both the apical-to-basolateral and basolateral-to-apical directions after cell cultures reached the confluence on parallel sets of Millicell well plates with similar TEER values (180 ± 11 Ω cm^2^).

The permeability (P_E_) values obtained by the diffusion studies indicated a high tendency of geraniol to permeate across the monolayer. In particular, the P_E_ value of the geraniol crossing from the basolateral to the apical compartments (B → A) was about 2⋅10^-3^ cm/min, similar to that shown by indomethacin on the same monolayer (1.5⋅10^-3^ cm/min) for the A → B transition (Ferretti et al., 2015). Indomethacin belongs to class II in the Biopharmaceutical Classification System (BCS), being a compound poorly soluble in water but capable of effectively permeating through biological membranes (Amidon et al., 1995), evidenced by a relatively high P_E_ value. Interestingly the A → B permeation profile of geraniol across the monolayer showed a P_E_ value of about 13⋅10^-3^ cm/min, an order of magnitude higher than that of indomethacin. As a consequence, the geraniol rate from the apical to basolateral compartments was roughly sixfold higher than its permeation in the opposite direction (*P* < 0.001). On the other hand, celiprolol, a drug known to be a substrate of the P-gp efflux transporter (Karlsson et al., 1993) expressed by NCM460 monolayers (Marchetti et al., 2016), showed an opposite behavior to that of geraniol, the celiprolol rate from basolateral to apical compartments being significantly higher than its rate in the opposite direction (Marchetti et al., 2016). The P_E_ values of geraniol, indomethacin and celiprolol were all obtained in our laboratory under the same experimental conditions using the same cell line and analytical instruments. These aspects therefore suggest an influx system for geraniol can actively transport geraniol from the intestinal lumen to the bloodstream. Moreover, a relatively high fraction of geraniol absorbed in the bloodstream can be assumed if it does not undergo degradation in the gastrointestinal tract.

In order to verify these hypotheses, geraniol was administered to rats by intravenous and oral routes. The drug’s plasma concentration profiles over time were obtained by analysis of blood samples withdrawn after the administrations. A liquid-liquid extraction procedure of geraniol from blood samples allowed accurate HPLC-UV analysis of its concentrations. The geraniol doses administered to rats were 50 mg/kg, one of the most widely used for the analysis of therapeutic effects following oral administration. In particular this dose was chosen since it is the lowest one previously employed for *in vivo* studies (Rekha et al., 2013, 2014; Cho et al., 2016; De Fazio et al., 2016).

*In vitro* measurements indicated that geraniol is highly stable in human and rat whole blood, whereas following intravenous administration geraniol is eliminated from the bloodstream with a relatively short half-life (about 12 min), starting from a concentration of about 300 μg/mL. This value (three times higher than the water solubility of geraniol at 25°C) is referred to the whole blood and not only to its plasmatic compartment. In fact, the liquid-liquid extraction method we used was applied on whole blood samples firstly hemolyzed and then deproteinated before the geraniol extraction in organic phase. As a consequence, despite the very low water solubility of geraniol (100 mg/L at 25°C), its concentration value of 300 μg/mL in whole blood can be attributed to the cellular and protein components able to increase geraniol concentration in the bloodstream. Indeed, the geometry and the hydrophilic/hydrophobic compensation of its molecule would allow geraniol a higher thermodynamic stability and permanence in a membrane bilayer model than other monoterpenes (Turina et al., 2006). This behavior is expressed through geraniol’s higher membrane/water partition coefficient (Pm/w) value (4.38) compared to other monoterpenes (2.44 for menthol; 3.99 for camphor; 3.37 for cineole) (Turina et al., 2006). Previous pharmacokinetic studies performed by us with very poorly water soluble drugs (such as rokitamycin – Gavini et al., 2011) or prodrugs (such as a conjugate of zidovudine with a bile acid – Dalpiaz et al., 2014), allowed to detect concentration values in whole blood up to 7 μg/mL following the intravenous administration of 1 mg/kg. These concentration values appear therefore in accordance with that found for geraniol, taking into account the administered doses.

Thus, the relatively short half-life of geraniol in the bloodstream can be attributed not only to fast metabolic and excretion processes, but also to a wide distribution in the body’s lipid compartments.

The profile of the geraniol concentrations in rat blood following oral administration of the emulsified formulation was characterized by a peak concentration at 30 min of about 270 μg/mL and an area under concentration (AUC) similar to that obtained by the intravenous administration of the same geraniol dose, indicating an absolute bioavailability of 92%.

These results indicate that the higher permeability of geraniol from the apical to basolateral compartments of the NCM460 monolayer with respect to the permeability in the opposite direction seems to be reflected in the intestinal barrier of rats, inducing a high bioavailability of geraniol in terms of both absorption rate and absorbed amounts. On the other hand, the very high bioavailability appears accompanied by a high elimination rate of geraniol from the blood, as found for intravenous administration. These data suggest that new formulations able to control the release of geraniol may be useful to obtain prolonged therapeutic effects of this compound. Moreover, the pharmacokinetic results indicate that appropriate formulations are needed to obtain therapeutic effects in the large intestine. In this regard, the geraniol adsorbed in vegetable fibers yielded an absolute bioavailability of 16%, indicating its ability to retain geraniol in the gut, reaching the colon where it has been demonstrated to elicit strong anti-colitic activity (De Fazio et al., 2016).

As geraniol was observed to induce neuroprotective effects (Rekha et al., 2013, 2014), its concentration in rat CSF was analyzed over time after the oral administration of the geraniol emulsified form in glycerol. Also in this case the concentration peak (about 2.5 μg/mL) was observed 30 min after oral administration, indicating that geraniol can directly permeate the central nervous system from the bloodstream. On the other hand, as observed in the bloodstream, the geraniol concentrations in rat CSF rapidly decreased over time. Taking these aspects into account, appropriate formulations could be designed for the nasal administration of geraniol to both enhance the amounts of geraniol targeting the central nervous system and to obtain prolonged therapeutic effects. Indeed, the nasal route has been shown to be efficacious for the brain targeting of neuroactive agents (Gomez et al., 2012; Tzabazis et al., 2017), namely when micro or nanoparticulate formulations obtained in the presence of absorption enhancers were used. In particular, nasal formulations based on chitosan have been demonstrated to show muco-adhesive properties, allowing a prolonged release of drugs in the central nervous system, thereby extending the therapeutic effect (Illum, 2012; Dalpiaz et al., 2015; Rassu et al., 2015).

The mild down-regulation exerted by geraniol on phase I enzymes (CYP3A1/2, CYP2E1, CYP1A2) might be of particular interest in pathological conditions as confirmed by Chen and coworkers (Chen et al., 2016) and Madankumar et al. (2013). In particular, Chen and coworkers showed how geraniol was able to ameliorate non-alcoholic steatohepatitis (NASH) induced by methionine-choline-deficient (MCD) diet in rats, also via CYP2E1 inactivation.

In mice, geraniol administration induced an increase in hepatic CAT, NQO1, SOD enzymatic activities, showing its antioxidant capacity on the liver. Many previous studies (Hasan and Sultana, 2015) confirmed this behavior and also proposed the mechanism: geraniol seems to be able to enhance Nrf2 protein expression and consequently activate Nrf2-directed antioxidant pathways (Xue et al., 2016). Moreover, a recent study demonstrated the key role of CYP induction in fat accumulation in non-alcoholic fatty liver disease, the advancement of steatotic liver to NASH and the relationship between these diseases and the high amount of reactive oxygen species (ROS) released (Abdelmegeed et al., 2012). In this scenario, the mild down-regulation exerted by geraniol (CYP3A1/2, CYP2E1, CYP1A2) associated with the increased anti-oxidant defenses might be of particular therapeutic interest. Even if animal models are widely used to predict the kinetics and toxicity of xenobiotics during Phase I and II metabolism studies in humans, none of the animal enzymatic systems are identical to their human counterparts. While our results give an initial idea of geraniol’s xenobiotic behavior, future *in vitro* (with human hepatocytes) or *in vivo* (with “humanized” mice) studies are needed to further support these preliminary findings.

## Conclusion

Permeation studies of geraniol across intestinal cell monolayers (NCM460) indicate the high tendency of this compound to permeate across the intestinal barrier at a rate roughly sixfold higher from the apical to basolateral compartments in comparison with the opposite direction. These results appear to agree with the high bioavailability of geraniol in terms of absorption rate (peak of blood concentration at 30 min) and absorbed amounts (absolute bioavailability of 92%) observed after its oral administration to rats. Geraniol appears able to permeate directly from the bloodstream to the central nervous system following its oral administration to rats, reaching detectable amounts in the CSF. Formulations obtained by absorption of geraniol on vegetable fiber led to drug retention in the gut (absolute bioavailability reduced to 16%) increasing geraniol’s efficacy in the colon as an anti-dysbiotic and anti-inflammatory agent.

Geraniol treatment was confirmed to increase anti-oxidative defenses in mice liver, with no signs of liver toxicity. Moreover, geraniol only slightly affected CYP enzyme activities. These results, in agreement with the findings of other studies (Ozkaya et al., 2017), demonstrate that treatment with geraniol can be considered safe even at high doses and for long periods of time.

## Author Contributions

AD, LM, and BP contributed to *in vitro* and HPLC experiments, including the acquisition, analysis, and interpretation of data. LF and SB performed experiments on rats including the acquisition, analysis, and interpretation of data. DC, MP, and FV performed analysis and interpretation of data on mice liver enzymes. AC and LDF grew mice, administered geraniol, and collected their livers and blood. MV and ES contributed to the conception and design of the work. ES drafted and revised the paper.

## Conflict of Interest Statement

The authors declare that the research was conducted in the absence of any commercial or financial relationships that could be construed as a potential conflict of interest.
